# Effects of Increased Nitrogen and Phosphorus Deposition on Offspring Performance of Two Dominant Species in a Temperate Steppe Ecosystem

**DOI:** 10.1038/srep40951

**Published:** 2017-01-19

**Authors:** Yang Li, Longyu Hou, Bing Song, Liuyi Yang, Linghao Li

**Affiliations:** 1 Xi’an Botanical Garden of Shaanxi Province, Institute of Botany of Shaanxi Province, Xi’an 710061, China; 2 State Key Laboratory of Vegetation and Environmental Change, Institute of Botany, Chinese Academy of Sciences, Xiangshan, Beijing 100093, China

## Abstract

Plants adapt to environment by plastic growth which will be transferred to offspring through transgenerational effect. Performance and response of maternal and offspring plant will affect population dynamics and community composition. However, it is scarcely understood how maternal nutrient environment affect the performance and response of offspring through transgenerational effect. Here we studied the impacts of nitrogen (N) and phosphorus (P) enrichment on maternal and offspring performances and responses of *Stipa krylovii* and *Artemisia frigida*. Seeds were collected from maternal plant experiencing N or/and P addition for three years in Inner Mongolia grassland. We found that maternal nutrient addition significantly affected seed traits, offspring biomass, and offspring responses of *A. frigida*. Maternal N addition significantly affected maternal reproductive biomass, seed traits of *S. kryloii*. Maternal P addition of *S. kryloii* significantly affected seed qualities, seedling biomass and seeding response to N addition. Our results suggested that transgenerational effects of N and P enrichment to the two dominant plant species existed in this ecosystem. Furthermore, the two species exhibited different adaptive strategies to future nutrient addition. These findings indicate that maternal environmental effect should be considered into the model projection of vegetation dynamics in response to ongoing environmental change.

With the increase of fossil fuel combustion and fertilization in agriculture, nitrogen (N) input into terrestrial ecosystem has been increased from 34 Tg N yr^−1^ to 100 Tg N yr^−1^, and it will reach to 200 Tg N yr^−1^ by 2050^1^. N deposition rate has increased substantially in China over the past two decades^2^, and it has reached 16 kg ha yr^−1^ in Inner Mongolia steppe ecosystems^3^. Meanwhile, phosphorus (P) becomes a limited element in most ecosystems because of N enrichment^4^. About 2/3 soil lacks P in China. P is either initially low or low because mineralization was limited by water and temperature in Inner Mongolia steppe ecosystems.

Both N and P are essential elements for plant growth. They could affect plant biomass accumulation, biomass allocation^5^, growth^6^, and seed quantity and quality^7^,^8^. Most previous studies focused on the effects of N and/or P addition on primary productivity^9^, soil carbon exchange^10^, soil microbe^11^, and plant community composition^12^,^13^,^14^. There was lack of study on how plant seeds and offspring respond to N and P addition and the possible subsequent influences on plant population dynamics and community composition^15^.

Plants may respond to environmental change by plastic growth. However, it is unlikely to yield significant insight into the future of population, community, ecosystem, and their feedbacks to environment change if only on the basis of vegetative plasticity alone. Plant response will become apparent after one or more generations. Furthermore, it will increase or decrease the initial phenotypic response to the environmental change^16^. Environment that parents experienced will play an important role on offspring response to changed circumstance^16^. Thus, it is essential to acquire detailed data about transgenerational effect for predicting plant response to future environmental change. Although it is possible that sexual reproduction may not play a significant role in the demography of long-lived perennial^17^, it is very important for maintaining genetic variability of population^18^ and saving population if they confront environmental change thus nature selection change.

Transgenerational effect may be an inheritance not based on DNA sequence^19^, which is frequently viewed as environmentally determined sources of phenotypic variation and has a substantial impact on the rate and direction of evolutionary change in response to directional selection^20^. It is well known that maternal environments, such as CO_2_^16^,^21^, water^22^,^23^, nutrients^5^,^24^, light^24^,^25^,^26^, and temperature^26^ substantially have effects on offspring development^27^. Moreover, transgenerational effect on the phenotype of progeny has been indicated in terms of the resource parents have available and will invest in offspring^28^. One example is that plants experienced N fertilized increased seed production, size and subsequent growth of their offspring^28^,^29^. Additionally, transgenerational effect would also take the form of transmitting information or chemical compounds to their offspring. For example, offspring of damaged plants increased the number of setose trichomes, a putative plant defense^30^. Maternal environmental conditions would also influence time of first flowering^22^,^31^ or physiological traits^32^, and so on. Furthermore, because transgenerational effect is species-specific, it may increase difficulty to forecast the community structure and biodiversity under environmental change. Therefore, more experimental studies are required to clarify whether and how maternal environment change affects offspring performance.

The most important thing about transgenerational effect on population is whether population would adapt to a novel and severe stress before being extinguished by it^33^ as many species especially plants are unlikely to migrate fast enough to track the rapidly changing climate^34^ or migration may be prevent by habitat fragmentation^22^,^34^. Adaptation is expected to rescue population being threatened with extinction^33^. For example, species under a five-year-drought could produce offspring with advancing first flowering and escape drought^22^. The adaptive response may play an important role in the initial stages of a population confronting a novel environment^27^ and the consequence of this response may not decrease over time^35^. However, previous studies always compared genotypes from different maternal environments when they grow in the same controlled offspring environment^28^. Although this way may distinguish clearly whether transgenerational effect exists, it may not easily to assess whether offspring adapt to changed environment, this remarkable, it is important for species which organism with limited dispersal. Offspring’s phenotype is the reflection of transgenerational effect and direct plastic response to immediate environment^25^,^27^,^28^. Therefore, the phenotype must be compared as same genotype growing in environment that is correlated with maternal condition, control environment or some conditions simulating future climate change.

To experimentally elucidate the mechanisms underlying the potential changes in maternal and filial performance attributes in response to N and P addition, a field experiment was conducted in a typical steppe in Inner Mongolian grassland. It is a part of a multi-factor field experiment initiated in April 2005^12^. Increased nutrients deposition, precipitation, and temperature have been documented to profoundly impact community composition and biodiversity in this steppe ecosystem^12^,^36^. *S. kryloii* and *A. frigida* are the two dominant species in the ecosystem. They coexist and compete to each other for space, nutrient, light, and other recourses. Their responses to environment change will induce changes in community composition and biodiversity in the ecosystem. The specific objectives of our study were addressed (1) how N and P addition and their interaction affect seed quantity and quality feature of *S. kryloii* and *A. frigida*, and (2) whether and how maternal environment will affect offspring growth and their responses to N and P addition, and (3) will the two dominant species adapt to future nutrient addition environments?

## Results

### Maternal reproductive biomass

**Figure 1:**
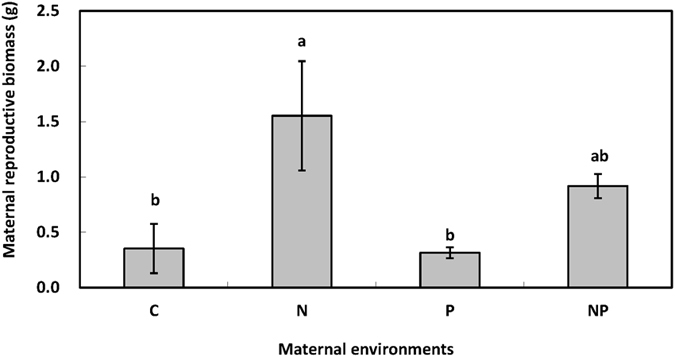
Maternal-treatment-induced changes in maternal reproductive biomass of *S. krylovii* (Mean ± 1 SE, n = 4). C: control treatment; N: nitrogen addition; P: phosphorus addition; NP: nitrogen and phosphorus addition in combine. Means with the same letter are not significantly different (*P* > 0.05).

Reproductive biomass of maternal plant was enhanced by 269.40% (F = 11.13, *P* < 0.05, df = 1, Fig. 1) under N addition in the maternal environment across the 2 maternal P addition treatments. P addition in the maternal environment decreased maternal biomass of *S. kryloii* by 35.19% (F = 1.54, *P* > 0.05, df = 1, Fig. 1). There was no interactive effect of maternal N addition and P addition on maternal biomass of *S. kryloii* (F = 1.22, *P* > 0.05, df = 1).

### Seed traits and their interdependence

#### Seed prodution

**Figure 2:**
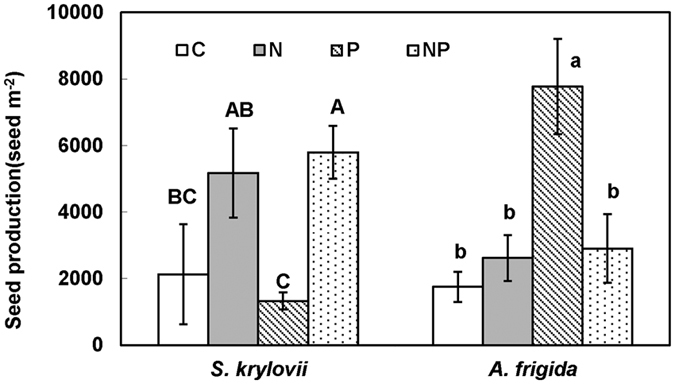
Maternal-treatment-induced changes in seed production (Mean ± 1 SE, n = 4). Means with the same uppercase letter for *S. krylovii* or lowercase letter for *A. frigida* are not significantly different (*P* > 0.05). See Fig. 1 for abbreviations.

**Figure 3:**
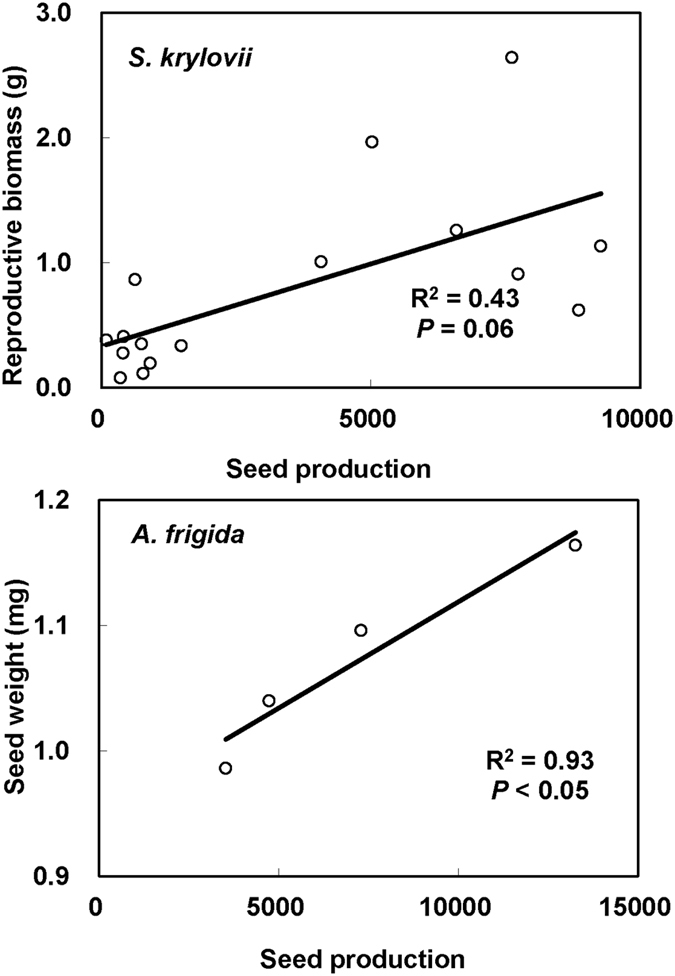
Dependence of seed production upon reproductive biomass of *S. Krylovii* (Y = 0.0001x + 0.3309) and dependence of seed weight upon seed production of *A. frigida* (Y = 2E-05x + 1.0003) across all the treatments.

Maternal N addition significantly increased seed production of *S. kryloii* by 217.66% (F = 11.86, *P* < 0.05, df = 1), and decreased seed production of *A. frigida* by 42.04% (F = 4.23, *P* = 0.06, df = 1, Fig. 2). Maternal P addition insignificantly decreased seed production of *S. kryloii* (F = 0.01, *P* > 0.05, df = 1), but significantly increased seed production of *A. frigida* by 144.34% (F = 10.49, *P* < 0.05, df = 1, Fig. 2). There was no interactive effect of maternal N addition and P addition on seed production of *S. kryloii* (F = 0.42, *P* > 0.05, df = 1). Maternal N and P addition played significant interactive effect on seed production of *A. frigida* (F = 8.69, *P* < 0.05, df = 1). For *A. frigida*, maternal N addition enhanced seed production by 49.51% and decreased it by 62.67% without and with P addition in maternal environment. Maternal P addition enhanced it by 343.92% and 10.85% without and with N addition in maternal environment, respectively. There was a significant correlation between reproductive biomass and seed production of *S. kryloii (r*^*2*^ = 0.42, *P* = 0.06, Fig. 3).

#### Seed mass

**Figure 4:**
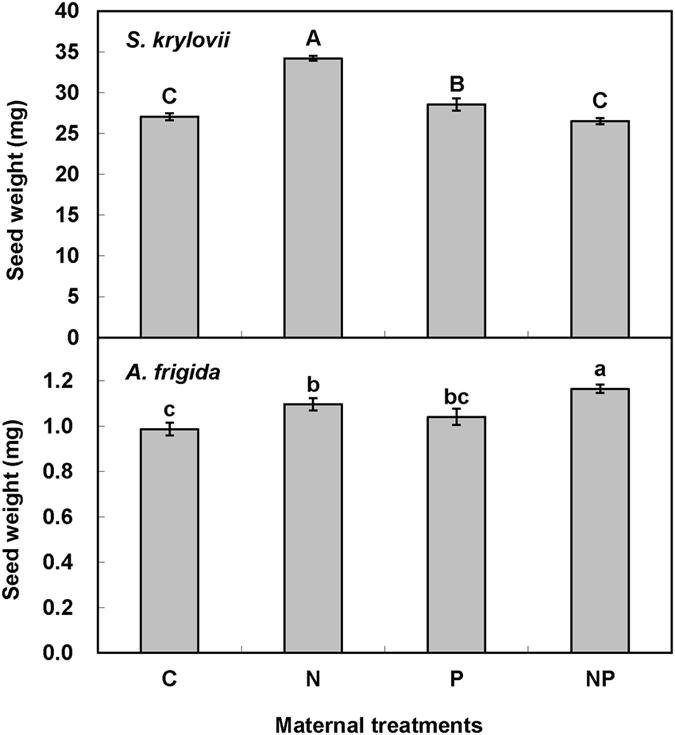
Maternal-treatment-induced changes in seed weight (mg/per seed, Mean ± 1 SE, n = 5). Means with the same uppercase letter for *S. krylovii* or lowercase letter for *A. frigida* are not significantly different (*P* > 0.05). See Fig. 1 for abbreviations.

Maternal N addition significantly increased seed mass of *S. kryloii* by 9.17% (F = 27.48, *P* < 0.001, df = 1, Fig. 4), and increased seed mass of *A. frigida* by 11.55% (F = 17.45, *P* < 0.001, df = 1, Fig. 4). Maternal P addition significantly decreased seed mass of *S. kryloii* by 10.10% (F = 40.45, *P* < 0.001, df = 1, Fig. 4), and increased seed mass of *A. frigida* by 5.86% (F = 4.74, *P* < 0.001, df = 1, Fig. 4). Maternal N and P addition played significant interactive effect on seed production of *S. kryloii* (F = 88.84, *P* < 0.001, df = 1). For *S. kryloii*, N-added in maternal environment increased seed mass by 26.40% and decreased it by 7.13% without and with P addition, respectively. P-added in maternal environment increased seed mass by 5.51% and decreased it by 22.47% in ambient and P addition environment (Fig. 4). There was no interactive effect of maternal N addition and P addition on seed mass of *A. frigida* (F = 0.06, *P* > 0.05, df = 1). There was a significant correlation between seed production and seed mass of *A. frigida (r*^*2*^ = 0.93, *P* < 0.05, Fig. 3).

#### Germination rate

**Figure 5:**
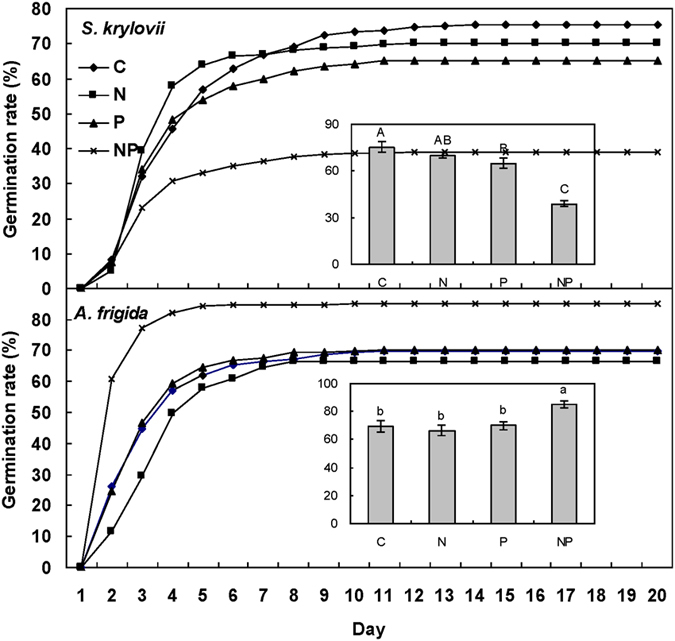
Maternal-treatment-induced changes in seed cumulative germination rate (%) and final germination percentage (inserts, means ± 1 SE, n = 5). Means with the same uppercase letter for *S. krylovii* or lowercase letter for *A. frigida* are not significantly different (*P* > 0.05). See Fig. 1 for abbreviations.

Maternal N addition significantly decreased seed germination rate of *S. kryloii* by 15.60 percentages (absolute difference, (F = 35.27, *P* < 0.001, df = 1, Fig. 5), and increased germination rate of *A. frigida* by 8.30 percentages (absolute difference, F = 3.07, *P* = 0.10, Fig. 5). Maternal P addition significantly decreased germination rate of *S. kryloii* by 20.80 percentages (absolute difference, F = 62.70, *P* < 0.001, df = 1), and increased germination rate of *A. frigida* by 9.60 percentages (absolute difference, F = 8.40, *P* < 0.05, df = 1). There was a significant interactive effect of maternal N addition and P addition on germination rate of the two species (for *S. kryloii*: F = 15.68, P < 0.05, df = 1; for *A. frigida*: F = 7.39, P < 0.05, df = 1). For *S. kryloii*, N-added in maternal environment decreased germination rate by 5.20 percentages and 26.00 percentages without and with P addition. P-added in maternal environment decreased germination rate by 10.40 percentages and 31.20 percentages in the ambient and P addition environment (Fig. 5). For *A. frigida*, N-added in maternal environment decreased germination rate by 3.20 percentages and increased it by 14.80 percentages without and with P addition. P-added in maternal environment increased germination rate by 0.60 percentages and 18.60 percentages in ambient and P addition environment (Fig. 5).

### Offspring biomass and response to environmental change

#### Offspring biomass

**Figure 6:**
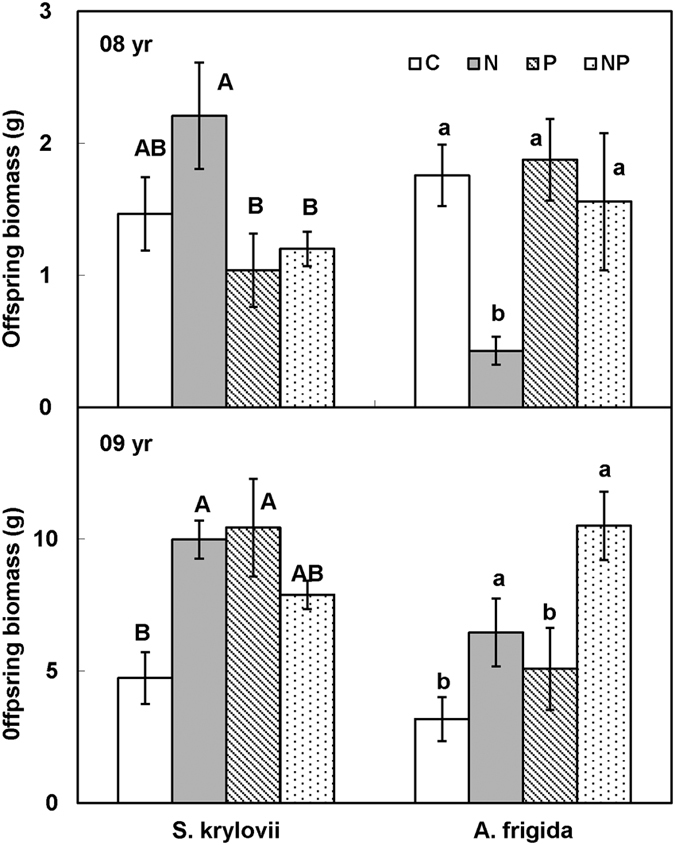
Maternal-treatment-induced changes in offspring biomass in 2008 and 2009 (g, Mean ± 1 SE, n = 7). Means with the same uppercase letter for *S. krylovii* or lowercase letter for *A. frigida* are not significantly different (*P* > 0.05). See Fig. 1 for abbreviations.

Maternal N addition did not significantly increased offspring biomass of *S. kryloii* in 2008 and 2009 (Fig. 6), but decreased offspring biomass of *A. frigida* by 50.66% (F = 9.68, *P* < 0.05, df = 1) in 2008, and increased offspring biomass of *A. frigida* by 105.23% (F = 11.70, *P* < 0.05) in 2009 (Fig. 6). Maternal P addition decreased offspring biomass of *S. kryloii* by 39.11% (F = 6.21, *P* < 0.05, df = 1) in 2008, and marginally increased offspring biomass of *S. kryloii* by 17.83% (F = 2.50, *P* > 0.05, df = 1) in 2009. Maternal P addition increased offspring biomass of *A. frigida* by 67.50% (F = 4.02, *P* = 0.06, df = 1), and 61.55% (F = 5.45, *P* < 0.05, df = 1) in 2008 and 2009, respectively. Maternal N and P played significantly interactive effect on offspring biomass of *S. kryloii* in 2009 (F = 11.63, *P* < 0.05, df = 1). Maternal N addition enhanced offspring biomass of *S. kryloii* by 110.55% and decreased it by 24.35% without and with P addition. Maternal P addition enhanced offspring biomass of *S. kryloii* by 120.04% in ambient treatment, and decreased it by 20.94% in N addition plot.

#### Offspring response

**Figure 7:**
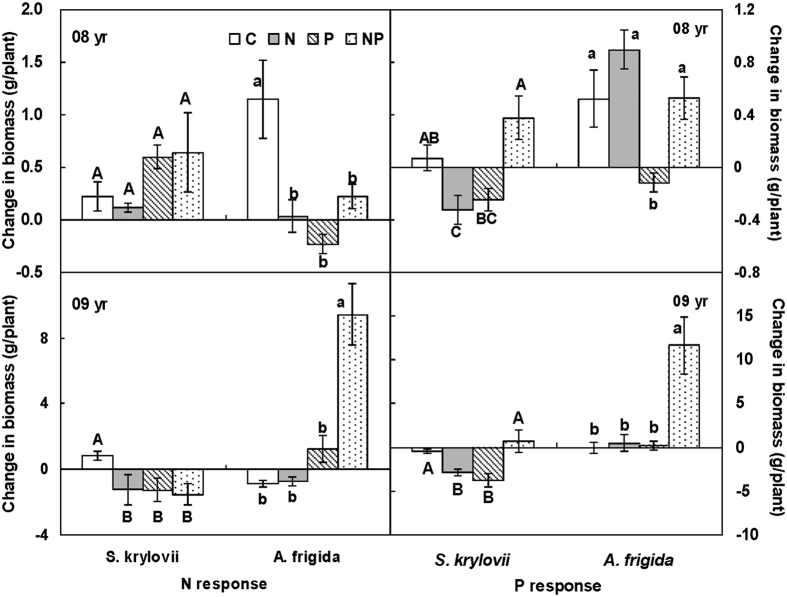
Maternal-treatment-induced changes in the N (a) and P (b) responses offspring in 2008 and in 2009 (Mean ± 1 SE, n = 7). Means with the same uppercase letter for *S. krylovii* or lowercase letter for *A. frigida* are not significantly different (*P* > 0.05). See Fig. 1 for abbreviations. Seedlings germinated by the seeds that collected from the 4 maternal treatments (control, N addition, P addition, and N and P addition in combine) were grown under the 4 offspring treatments. The N responses were calculated as the difference in offspring biomass growing with and without N addition in the offspring environment. The N responses were calculated as the difference in seedling biomass between the ambient and increased P in the offspring environment.

In 2008, maternal N addition did not significantly affect seedlings responses of *S. kryloii* to offspring N addition (F = 0.02, *P* > 0.05, df = 1, Fig. 7), while maternal P addition significantly increased it by 257.95% (F = 4.51, *P* < 0.05, df = 1, Fig. 7). Although maternal nutrient addition environment had no effects on seedlings responses of *S. kryloii* to offspring P addition (maternal N addition: F = 0.97, *P* > 0.05, df = 1; maternal P addition: F = 2.51, *P* > 0.05, df = 1), maternal N and P addition played a significant interactive effect on it (F = 18.33, *P* < 0.001, df = 1). Maternal N addition decreased seedlings responses of *S. kryloii* to offspring P addition in ambient and P addition environments by 551.08% and 251.34%, respectively. Maternal P addition decreased seedlings responses of *S. kryloii* to offspring P addition in ambient and N addition environments by 449.00% and 217.09%, respectively (Fig. 7).

In 2008, maternal N addition did not affect seedlings responses of *A. frigida* to offspring N addition (F = 2.40, *P* > 0.05, df = 1, Fig. 7), maternal P addition significantly decreased it by 100.60% (F = 7.73, *P* < 0.05, df = 1, Fig. 7). There was an interactive effect of maternal N and P addition on seedlings responses of *A. frigida* to offspring N addition (F = 13.47, *P* < 0.05, df = 1). Maternal N addition decreased seedlings responses of *A. frigida* to offspring N addition in ambient and P addition environments by 97.09% and 196.30%, respectively. Maternal P addition decreased it by 120.06% without maternal N addition and increased it by 563.83% with maternal N addition. Maternal N addition significantly enhanced seedlings responses of *A. frigida* to offspring P addition by 248.95% (F = 10.40, *P* < 0.05, df = 1), while maternal P addition significantly decreased it by 70.96% (F = 10.23, *P* < 0.05, df = 1). There was no interactive effect of maternal nutrition addition on seedlings responses of *A. frigida* to offspring P addition (F = 0.74, *P* > 0.05, df = 1, Fig. 7).

In 2009, maternal environments effects on offspring responses were different from seedlings in 2008 because plants of *S. kryloii* and *A. frigida* began to mature (Fig. 7). To *S. kryloii*, both maternal nutrient addition marginally increased offspring responses to offspring N addition environments by 567.92% (F = 2.92, *P* > 0.05, df = 1, Fig. 7) and 581.40% (F = 2.96, *P* = 0.10, df = 1, Fig. 7), respectively. There was no interactive effect of maternal nutrient on offspring responses to offspring N addition (F = 1.65, *P* > 0.05, df = 1). Maternal N (F = 1.71, *P* > 0.05, df = 1) and P (F = 0.03, *P* > 0.05, df = 1) addition environments did not affect offspring responses to offspring P addition. There was an interactive effect of maternal N and P addition on offspring responses to offspring P addition of *S. kryloii* (F = 20.34, *P* < 0.001, df = 1). Maternal N addition increased offspring responses to offspring P addition of *S. kryloii* by 560.00% in ambient environment, and decreased it by 118.66% in maternal P addition. Maternal P addition increased offspring responses to offspring P addition of *S. kryloii* by 759.04%, and decreased it by 124.29% in maternal N addition.

In 2009, to *A. frigida*, maternal nutrient environments significantly altered offspring responses to offspring N addition environments by 2610.00% (F = 16.43, *P* < 0.001, df = 1, Fig. 7) and −748.29% (F = 35.47, *P* < 0.001, df = 1, Fig. 7), respectively. Maternal nutrient addition environments had interacted effects on offspring responses to offspring N addition of *A. frigida* (F = 15.32, *P* < 0.001, df = 1). Maternal N addition decreased offspring responses to offspring N addition by 16.04%, and increased it by 677.80% without and with maternal N addition. Maternal P addition decreased offspring responses to offspring N addition by 236.05% and 1360.27% without and with maternal P addition, respectively. Maternal nutrient addition increased offspring responses of *A. frigida* to offspring P addition environments by 7806.49% (F = 11.87, *P* < 0.05, df = 1) and 2306.10% (F = 10.57, *P* < 0.05, df = 1). Maternal nutrient addition environments had interacted effects on offspring responses to offspring P addition of *A. frigida* (F = 9.77, *P* < 0.05, df = 1). Maternal N addition decreased offspring responses to offspring P addition of *A. frigida* by 1836.39%, and increased it by 6159.65% without and with P addition. Maternal P addition decreased it by 716.81%, and increased it by 21213.60% in ambient and in N addition environment (Fig. 7).

## Discussion

To some extent, maternal nutrient addition played important roles on seed traits, offspring performance and responses to future nutrient addition circumstance in the present study. Maternal nutrient addition was in favor of the two species adapting to future nutrient addition environment.

### Impacts of maternal nutrient environments on seed attribute

Seed performance of *S. kryloii* and *A. frigida* after dispersal were highly dependent on maternal environmental cues in our study, which were in line with previous studies^23^,^25^,^26^. Nutrient addition will alter maternal plant growth^6^, photosynthesis^37^, and root extend^38^ to stimulate biomass accumulate especially reproductive biomass allocation^29^. Significant correlation in reproductive biomass and seed production of *S. kryloii* in our study confirmed reproductive biomass usually has direct positive effect on seed number and/or seed mass. Besides, significantly correlation between seed production and seed mass of *A. frigida* confirmed the tradeoff between seed number and mass^28^. Although seed mass usually has positive effect on seed germination, there was no significantly correlation between seed germination and seed mass of *S. kryloii* and *A. frigida* in this study. Because seeds of the two species were too small to affect germination, or germination of the two species depended on environmental factors or signals^39^.

Although there was a significant increased in seed mass of *S. kryloii*, seed germination percentage was not increased consequently when maternal plants experienced N addition in our study. Firstly, N addition increased seed mass via increased mass of accessory structure such as seed coat or awn rather than cotyledon or endosperm which affects directly seed germination^24^. In our study, maternal N-addition stimulated awn length by 12.9% (Li Y, unpublished data) and could subsequently to enhance seed mass. Seed with longer awn might be beneficial in dispersing and avoiding competition. Secondly, we investigated ratio of nutrient tiller numbers to reproductive till numbers of maternal plants in field in 2008. N addition stimulated the ratio from 1.65 to 3.79 (Li Y, unpublished data). It indicated nutrient growth is superior to reproduction growth. *S. kryloii* invested more resource to nutrient growth under N addition condition. During the procedure of seed forming, nutrient growth acquitted more resource through competition. Thus *S. kryloii* could produced bigger seed but with little resource to support germination. Our result was consistent with that seed size has no effect on germination percentage^24^. Thirdly, maternal N addition increased offspring biomass, which indicated that survival seedlings were more vitality. It confirmed previous studies^22^,^40^ that bigger seed increased seedling performance. Impact of maternal N addition resulted notable intraspecific variation of seed size^29^. It meant that big seeds become bigger and small seeds become smaller. The proportion of empty seeds could increase. The germination percentage declined because the number of big seeds was less than the number of small seeds, and the seeds were small enough to hamper germination. The reproductive strategy ensures that more resource is invested into less but vitality seeds^40^. For plant population, the tradeoff is meaningful to ensure population generation in environments where mortality is high due to resource limitation or competition^41^. It may maximize offspring survival and colonize to new favorable sites^42^. Furthermore, seed from maternal N addition environment germinated more quickly according to our study. Rapid germination is an advantage in intense competition environment and in boreal area^43^. Earlier germination may give plants more chance to exploit the short growing season. Furthermore, earlier seedlings give more stress to later seedlings through space or resource competition. The interspecific relationship change consequently, and so do community composition and biodiversity^7^. Meanwhile, increased seed provisioning (seed quantity and quality) can promote offspring’s success through reduction of time required for immediate plastic response to an environmental challenge and expression of extreme developmental response^33^. Our study also suggested that maternal environment did not affect seed traits equally. The absence of true plasticity in beneficial traits can be compensated for by true plasticity in compensating traits^44^.

According to our observations, *S. kryloii* and *A. frigida* invested more resource to increased seed production rather than seed mass. Their reproductive strategies are *R*-strategy. The ability for prolific seed production is meaningful under fluctuant and stressful circumstances^45^. More numerous seeds have an advantage in fecundity^46^, and might be better at dispersing and a consequently higher probability that seeds colonize new favorable habitats^47^. Furthermore, if seeds were decay, or drop in unsuitable places to germination, or were consume by predators, population still reserve enough seeds to establishment because of higher seed production^48^. Therefore, plant responds to changes in resource supply by a change in seed number rather than mass^49^ ensured reproduction success of population. In our study, *S. kryloii* and *A. frigida* utilized different nutrient to increased seed production. It may be one of mechanisms of the two dominant species coexisting in the ecosystem.

### Impacts of maternal nutrient environments on offspring performance

It is doubtless that N addition can influence the growth of various plants^50^. However, maternal N addition played more positive effects on *A. frigida* than on *S. kryloii*; furthermore, the N-added effects on *A. frigida* would become more positive over time in our study. According to previous studies in the region, long-term N addition leads to loss of species richness via enhancing and suppressing biomass of *S. kryloii* and *A. frigida*, respectively^12^,^13^. *A. frigida* may be gradually replaced by *S. kryloii* in response to N addition. It seems to opposite to our results that the maternal effects may amplify *A. frigida* growth, and the maternal effects were insignificant for *S. kryloii*. There were two reasons. First, greenhouse experiment was different from field observations, because greenhouse experiment in our study was not taken interspecific competition for nutrient between the two species into account. Interspecific competition was one of the most important factors that affect community composition. Second, N addition played important roles on population of *S. kryloii* via increased seed production. It was an advantage to occupy more habitats in ecosystem. Higher plant of *S. kryloii* grew fast to occupy upper space, and intercept more light. Lower-canopy plant *A. frigida* had to suffer from lower light availability condition and to constrain its photosynthesis^51^. *A. frigida* grew smaller and *S. krylovii* occupied more proportion in community with N availability increasing. Thus, it is an explanation to previous results that loss of species richness under long-term N addition treatments.

P addition in maternal environment played different roles on offspring biomass of *S. kryloii* and *A. frigida* in our study. On the one hand, according to previous study, P addition advanced the flower time of *S. kryloii* by 5–15d, and it delayed the flower time of *A. frigida* by 10–15d in the ecosystem. *S. kryloii* flowered in July and August when most species flowered. *A. frigida* flowered in September and October when most species had finished growth. Thus, advanced flower timing of *S. kryloii* was a competition for nutrient and light to other species, which can decrease biomass and allocation to reproduction. Seed and seedling traits may decrease consequently. *A. frigida* avoided competition to other species because of delay flower timing of *A. frigida*. On the other hand, *A. frigida* seedlings required higher external P level for their maximal growth than those of *S. kryloii*^52^. Thus, P addition in our field experiment may exceed requirement for maximal growth of *S. kryloii*, although it stimulated growth of *A. frigida*.

Nutrient addition in maternal environment had different effects on seedlings (08 yr) and mature plant (09 yr) of the two species. Plants were planted in pot, and soil nutrient decreased with plants growth. The nutrient limitation became more and more serious when mature plant began reproduction. Plants that parents experienced nutrient addition could grow better in infertile soil. It proved transgenerational effect do exist. Furthermore, transgenerational effect is not only simply “amplifies” or “diminishes” maternal plastic responses to environment change, but also improves offspring adaption to environment change^43^.

### Species adapt to future nutrition environment through transgenerational effects

Our results supported the hypothesis that transgenerational effects do exit between maternal plants and offspring^24^,^43^. Furthermore, maternal plant transfers advantage to their offspring^31^. Offspring exhibited different life history traits if their maternal plants experienced different environments through transgenerational effects whatever based on genetic changes^19^,^22^,^24^.

Our study indicated that plants not only endures environmental change, but also take plastic phenotypic change to adaptive environmental change^25^,^33^,^42^ according to offspring responses. This is inconsistent with some previous studies^35^ that found maternal effects reducing the positive effects of changed environment^21^. Our seed, seedling, mature offspring plant, and responses results suggest that the two species *S. kryloii* and *A. frigida* will growth better under future N and/or P addition circumstance. This adaptation to environmental change may be especially important for the persistence of plants, because limited dispersal ability and habitat fragmentation may prevent plants from migrating^22^,^34^. Estimate of growth or other traits increased based on short-term plastic growth responses may underestimate the magnitude of nutrient addition effects. Our findings have important implications for prediction future ecosystem consequence; in that species adaptation should be taken into consideration to project the species composition responses to future environmental change.

## Conclusions

As two dominance species, the performance of seed and offspring of *S. kryloii* and *A. frigida* are important to community composition and biodiversity in the semi-arid temperate steppe of northern China. Our results demonstrated that (1) maternal nutrient addition significantly affected seed traits of the two species. Maternal effects were not equal among these traits, because there were a tradeoff and compensation. (2) Maternal nutrient addition affected offspring performance and responses to future nutrient addition circumstance to some extent. Maternal nutrient addition played important roles on offspring biomass and responses of *A. frigida*. Maternal nitrogen addition significantly affected offspring response to P addition of *S. kryloii*. And maternal P addition significantly affected seedling biomass of *S. kryloii*. To some extent, N and P addition played interactive effects on seed quality and offspring biomass of the two species. (3) Maternal nutrient environments did affect offspring performance through maternal effects in the ecosystem according to our study. Furthermore, the two populations may remain constant or rise with increase of N and/or P.

However, the different responses of seed and offspring traits of the two species to nutrient addition made it difficult to predict the future community composition and biodiversity in this region under N and P addition regimes. Further studies on competition between the two species which parents experiencing nutrient addition are needed. Moreover, recent studies propose that many phenotypic expressions are size-dependent processes. It can eliminate size-dependency and leave only the environmental-induced plasticity through a size-correction to the reaction norm^44^. Thus, size-dependent plasticity must be taken into account in our future studies.

## Methods

### Study site

Our study was conducted at the Duolun Restoration Ecology Station of Institute of Botany, Chinese Academy of Sciences (42°02′N, 116°17′E, 1324 m asl), Inner Mongolia Autonomous Region of China. Its mean annual temperature is 2.1 °C with monthly mean temperatures of 18.9 °C in July and −17.5 °C in January. Mean annual precipitation is 385.5 mm with approximately 80% concentrated from June to September. The soil in this area is classified as chestnut (Chinese classification) or Calcic Luvisols according to the FAO classification with 62.75 ± 0.04% sand, 20.30 ± 0.01% silt, and 16.95 ± 0.01% clay, respectively. Soil bulk density and pH values are 1.31 ± 0.02 g cm^−3^ and 6.84 ± 0.07, respectively. Soil organic C and total N contents are 16.10 ± 0.89 and 1.48 ± 0.10 g kg^−1^, respectively. Vegetation in the area is a typical steppe community and dominated by perennials, including *Stipa krylovii* Roshev., *Artemisia frigida* Willd., *Potentilla acaulis* L., *Potentilla tanacetifolia* Willd., *Cleistogenes squarrosa* (Trin.) King, *Allium bidentatum* Fisc. ex prokh., and *Agropyron cristatum* (L.) Gaertn.

### Plant material

*S. krylovii* and *A. frigida* that coexist in the temperate grassland in northern China were selected. *S. krylovii,* a perennial grass, is a widespread and predominant species in the arid and semi-arid grassland in Inner Mongolia, China. *S. krylovii* is tall bunchgrass and its vegetative and reproductive tillers can grow up to 50 cm in height at the peak of the growing season. It flowers in last July and August. Seed dispersal takes place in September. *A. frigida* is a semi-shrub, and occupies 38% of total foliar biomass in this grassland. Its vegetative tiller is usually less than 10 cm and its reproductive tiller can grow up to 30 cm in height. It flowers in last August. Seed dispersal takes place in October. As investigation in August 2008, density of *S. krylovii* is approximately 33 individuals per square meter, including 208 vegetative tillers and 126 reproductive tillers. Density of reproductive tillers for *A. frigida* is approximately 14 individual per square meter.

### Field experimental design

*In situ* observations were conducted at the permanent site of the Duolun Global Change Multifactor Experiment (GCME) that established in 2005. We chose four 92 × 60 m area. Each area was divided into four 44 × 28 m plots. Each plot was randomly assigned to one of the four nutrient treatments, including the control (C, no nutrient addition), nitrogen addition (N, 10 g N m^−2^year^−1^), phosphorus addition (P, 5 g P m^−2^year^−1^), and addition of both nitrogen and phosphorus (NP). We applied urea in 2005 and NH_4_NO_3_ in 2006–2009 in N addition treatments, and applied calcium superphosphate in P addition treatments. Nutrient addition were applied once a year in middle July.

### Measurements of vegetation and seed attributes

We selected randomly 1 × 1 m^2^ quadrate in each plot in field in September 2008, resulting in total of 16 quadrates. Numbers of plants, vegetative tillers and reproductive tillers were observed in each quadrate. We collected aboveground biomass of *S. krylovii* and *A. frigida* at their maturity stage, respectively. Three reproductive tillers for *A. frigida* in each quadrate were used to calculate fruit numbers of one reproductive tiller. Ten reproductive tillers for *S. krylovii*, three fruits for *A. frigida* in each quadrate were used to calculate seed numbers in one fruit or reproductive tiller. Then we used product of number of reproductive tiller in 1 m^2^, number of fruit in a reproductive tiller and number of seed in a fruit to calculate seed production for *A. frigida*. For *S. krylovii*, seed production was product of number of reproductive tiller in 1 m^2^ and number of seed in a reproductive tiller. Mature seeds were collected in each plot and were air-dried until the next growing season. In order to test maternal environment effects on seed size, 500 mature seeds were selected randomly to measure germination percentage before measuring seeds weight for each plot. We used an electronic balance with the accuracy of 0.1 mg to weight 100 of the 500 seeds to determine the mean seed mass. 100 seeds were placed on moist filer paper in a sealed dish and germinations were observed every day in an incubating oven with 25 °C constant temperature.

### Greenhouse observations

We established a greenhouse to observe effects of maternal environment on offspring beside field studies in Duolun Count in summer 2008. In September and October 2007, we collected seeds of *S. krylovii* and *A. frigida* from nutrient addition plots in GCME. Seeds were air-dried, and were first germinated in ambient environment. After 15 days, germinate seedlings of 4 maternal treatments (control, N addition, P addition, and N and P addition in combine) were transplanted into pots which kept in the greenhouse in the middle of May. The seedling environments were produced in 15 cm × 15 cm plant bands containing a mixture of soil from field near field studies site. At the end of May, the pots together with seedlings were assigned to 4 offspring treatments including control, N addition, P addition and N and P addition in combine with 14 replicates for each treatment according to a full-factorial design. Each pot was watered to maintain growth. Beginning in late May, 100 ml of 0.5 gL^−1^ NH_4_NO_3_ solution was added to each N addition alone as well as the N plus P plot for 10 times at a 10-day interval during the growing season in one year. 100 ml of 0.4 gL^−1^ KH_2_PO_4_ solution was added to each P addition alone as well as the N plus P plots at the same frequency. The two solutions were not added at the same day. Instead of solution, the control plots were added 100 ml water at solution-added-day. Plants were harvested in late August, 2008 and 2009, respectively. The dry mass was oven-dried at 65 °C for 48 h to constant weight.

### Statistical analyses

Two-way ANOVAs with a full factorial design were used to examine the main and interactive effects of N and P on maternal biomass, seed production, mass, germination percentage, offspring biomass, and offspring responses. Linear regressions were used to examine the relationships of seed mass with seed production and seed germination percentage with seed mass. There were 4 replications for maternal biomass and seed production, 5 replications for seed mass and germination percentages, and 7 replications for offspring biomass and responses. All the above statistical analyses were conducted with SAS software (SAS Institute Inc., Cary, NC, USA).

## Additional Information

**How to cite this article**: Li, Y. *et al*. Effects of Increased Nitrogen and Phosphorus Deposition on Offspring Performance of Two Dominant Species in a Temperate Steppe Ecosystem. *Sci. Rep.* **7**, 40951; doi: 10.1038/srep40951 (2017).

**Publisher's note:** Springer Nature remains neutral with regard to jurisdictional claims in published maps and institutional affiliations.
